# Integration of Spatial Information Increases Reproducibility in Functional Near-Infrared Spectroscopy

**DOI:** 10.3389/fnins.2020.00746

**Published:** 2020-07-28

**Authors:** Sergio Luiz Novi, Edwin Johan Forero, Jose Angel Ivan Rubianes Silva, Nicolas Gabriel S. R. de Souza, Giovani Grisotti Martins, Andres Quiroga, Shin-Ting Wu, Rickson C. Mesquita

**Affiliations:** ^1^“Gleb Wataghin” Institute of Physics, University of Campinas, Campinas, Brazil; ^2^Brazilian Institute of Neuroscience and Neurotechnology, Campinas, Brazil; ^3^School of Electrical and Computer Engineering, University of Campinas, Campinas, Brazil

**Keywords:** fNIRS, test–retest, neuronavigation, reproducibility, within-subject analysis, data analysis

## Abstract

As functional near-infrared spectroscopy (fNIRS) is developed as a neuroimaging technique and becomes an option to study a variety of populations and tasks, the reproducibility of the fNIRS signal is still subject of debate. By performing test–retest protocols over different functional tasks, several studies agree that the fNIRS signal is reproducible over group analysis, but the inter-subject and within-subject reproducibility is poor. The high variability at the first statistical level is often attributed to global systemic physiology. In the present work, we revisited the reproducibility of the fNIRS signal during a finger-tapping task across multiple sessions on the same and different days. We expanded on previous studies by hypothesizing that the lack of spatial information of the optodes contributes to the low reproducibility in fNIRS, and we incorporated a real-time neuronavigation protocol to provide accurate cortical localization of the optodes. Our proposed approach was validated in 10 healthy volunteers, and our results suggest that the addition of neuronavigation can increase the within-subject reproducibility of the fNIRS data, particularly in the region of interest. Unlike traditional approaches to positioning the optodes, in which low intra-subject reproducibility has been found, we were able to obtain consistent and robust activation of the contralateral primary motor cortex at the intra-subject level using a neuronavigation protocol. Overall, our findings support the hypothesis that at least part of the variability in fNIRS cannot be only attributed to global systemic physiology. The use of neuronavigation to guide probe positioning, as proposed in this work, has impacts to longitudinal protocols performed with fNIRS.

## Introduction

Functional near-infrared spectroscopy (fNIRS) has emerged as a promising tool to measure brain function over the years (Ferrari and Quaresima, 2012; Boas et al., 2014; Strait and Scheutz, 2014; Herold et al., 2018; Quaresima and Ferrari, 2019). fNIRS relies on the fact that near-infrared light (∼700–900 nm) is absorbed by oxy- (HbO) and deoxy-hemoglobin (HbR), which allows one to estimate HbO/HbR concentration changes in biological tissue. In the brain, changes in HbO and HbR reflect (at least partially) neural activity due to neurovascular coupling (Obrig et al., 2002; Devor et al., 2005; Rovati et al., 2007; Mesquita et al., 2009; Ou et al., 2009; Phillips et al., 2015). Due to its high temporal resolution, portability, and versatility, fNIRS has become a suitable choice to investigate brain function non-invasively over a variety of populations, ranging from neonates to adults, both in the healthy and in the diseased brain (Tsuji et al., 2000; Brown et al., 2002; Terborg et al., 2004; Kim et al., 2010, 2014; Dehaes et al., 2015; de Oliveira et al., 2017, 2019; Forero et al., 2017; He et al., 2018; Forti et al., 2019). More recently, wearable technologies have expanded fNIRS protocols to study activities in natural and unconstrained environments (Piper et al., 2014; McKendrick et al., 2016; Pinti et al., 2018). These novel technologies afford new application scenarios never thought before, such as learning, training, and rehabilitation (Hatakenaka et al., 2007; Sitaram et al., 2009).

The reliability of fNIRS to answer questions in several fields, however, depends highly on the reproducibility of the results derived from the optical signal. From a scientific perspective, reproducibility is one of the cores of progress. Test–retest protocols have been previously applied to investigate the variability of fNIRS data. Due to the increased interest of the neuroscience community in functional connectivity, several studies have attempted to characterize the reliability of resting-state networks extracted from the fNIRS signal during the resting state (Mesquita et al., 2010; Zhang et al., 2011; Niu et al., 2013; Novi et al., 2016). Zhang et al. (2011) concluded that fNIRS connectivity maps are highly robust at the group level and fairly reliable at the individual level. Niu et al. (2013) and Novi et al. (2016) independently showed that graph-theoretical approaches can extract reliable features from fNIRS data despite greater intra-individual variability in functional connectivity experiments.

The reproducibility of fNIRS data in functional protocols has also been investigated. Most of the studies agree that the fNIRS signal is reproducible over group analysis, while within-subject fNIRS reproducibility has been shown to be poor (Watanabe et al., 2003; Kono et al., 2007; Plichta et al., 2007; Schecklmann et al., 2008; Kakimoto et al., 2009; Blasi et al., 2014; Dravida et al., 2017). By longitudinally measuring infants, Blasi et al. (2014) observed that HbO concentration changes were highly reproducible over group analysis yet just acceptable within subjects. More recently, Wiggins et al. (2016) analyzed speech protocols on healthy adults and found that the fNIRS signal is highly variable within subjects during speech-based protocols.

Several aspects related to the fNIRS technique can contribute to the increase in variability. Previous studies have suggested that systemic physiology plays an important role in the fNIRS signal (Joseph et al., 2006; Kirilina et al., 2012; Caldwell et al., 2016). Motion artifacts can also influence fNIRS results if not properly corrected (Huppert et al., 2009a; Cooper et al., 2012; Brigadoi et al., 2014; Novi et al., 2020). In addition, lack of spatial information may also be an important factor to decrease subject reproducibility. In typical fNIRS experiments, fNIRS optodes are placed on the scalp of the volunteers with little known information regarding the brain structure below the surface. To maximize accuracy in fNIRS measurements, one often decides to cover a broad surface with as many sources and detectors as possible. The 10–20 system localization (Klem et al., 1958) has also been widely used in fNIRS protocols, but this procedure is subjected to systematic errors of measurement and does not account for subject variation. Currently, the use of fNIRS probes with standard layouts in fixed positions is the best procedure to minimize spatial variation across multiple measurements.

In the present work, we hypothesized that the lack of precise anatomical information could contribute to a further increase in the within-subject variability in fNIRS. To test our hypothesis, we devised a protocol consisting of two sequences of measurements: with and without a real-time neuronavigation system to guide the placement of the fNIRS optodes. We assessed within-subject reproducibility by performing motor stimulation in three to five independent sessions in 10 subjects, both at the same day and in subsequent days. Extra-cortical contributions and systemic physiological parameters [i.e., heart rate (HR) and arterial blood pressure] were also included in the analysis.

## Materials and Methods

### Subjects and Experimental Protocol

We acquired data from 10 young healthy male, right-handed volunteers with ages ranging from 19 to 34 years [mean (standard deviation) age = 22 (5) years]. We excluded subjects from the study when they presented any history of neurological or vascular disease, magnetic resonance imaging (MRI) contraindications (e.g., metal implants), or lack of a palpable inion because of neuronavigation constraints (see section “Real-Time Neuronavigation”). All participants provided signed informed consent prior to the experiment protocol. The study protocol is in accordance with the Declaration of Helsinki, and it was approved by the local Ethics Committee at the University of Campinas, where the experiments were carried out.

All subjects were required to perform the same experimental protocol at different times (i.e., sessions). For each session, participants performed a right-hand finger-tapping block-designed protocol consisting of 30 blocks of 2-s stimulation interleaved with a rest period that varied between 10 and 20 s. [Note that the rest period was randomized to decrease the probability of task synchronization with periodic physiological noise, such as Mayer waves (Yücel et al., 2016)]. Since we wanted to investigate the effect of spatial information on the fNIRS reproducibility, we attempted to minimize the effects of circadian cycle in the measurements. Therefore, we acquired three independent sessions at the same time of the day over three different days for all subjects. For five of the 10 subjects, we additionally performed fNIRS measurements in three different periods of the day in one of the 3 days. In the latter case, measurements were taken in the morning (∼9 a.m.), in the afternoon (∼2 p.m.), and in the evening (∼6 p.m.). This approach allowed us to investigate how differences in systemic physiology (induced by different times of the circadian cycle) affect fNIRS reproducibility. In all sessions, we independently measured the subject’s HR and arterial blood pressure with a commercial automated blood pressure monitor before and after the experimental protocol.

### fNIRS Signal Acquisition

**FIGURE 1:**
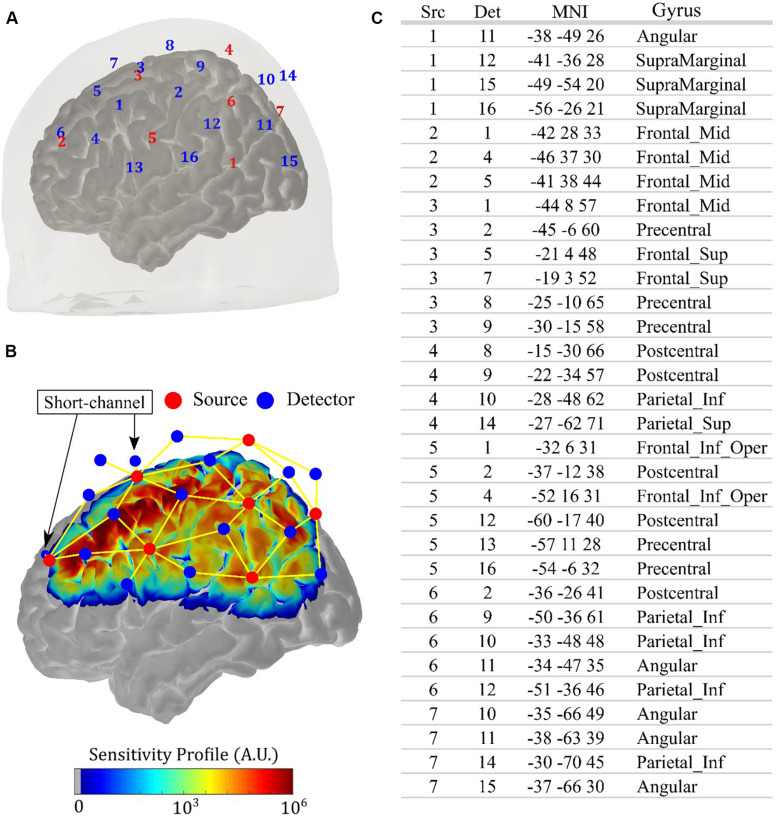
Details of the anatomical positioning of the fNIRS probe. **(A)** Source-detector configuration of the optical probe used in the study, with 14 sources (red) and 32 detectors (blue), allowing 64 channels with source-detector separation of 3 cm and four short channels with source-detector separation of 0.8 cm. Since the probe is symmetric, the right hemisphere was omitted in the figure. **(B)** Sensitivity profile of the optical probe on the left hemisphere obtained with Monte Carlo simulation, considering all channels (yellow lines). The position of the short channels is indicated by the arrows. **(C)** Projections of the channels onto the brain cortex, with MNI coordinates and the associated gyrus, following the specifications of source (Src) and detector (Det) shown in **(A)**.

All measurements were performed with a commercial continuous-wave (CW) NIRS system (NIRScout, NIRx Medical Systems, New York, NY, United States). We designed an optical probe with 14 sources (each source has two LEDs centered at 760 and 850 nm, ∼5 mW light power emission each) and 32 detectors, allowing 64 source-detector combinations (i.e., channels) at 3 cm and four source-detector pairs (i.e., short-channels) at 0.8 cm. This setup allowed an acquisition frequency of 8.9 Hz. We fixed the optodes with a 10–20 standard head cap. Figure 1A shows the location of each source and detector for an arbitrary volunteer. The optodes were placed symmetrically with respect to the brain hemispheres, and the probe was designed to be sensitive to the primary and secondary motor cortices, covering most of the parietal and the frontal lobes. Figure 1B shows the sensitivity profile for the optical setup, which was obtained with Monte Carlo simulations via the AtlasViewer package (Aasted et al., 2015). The position of the short channels is also highlighted in Figure 1B; they were located in the frontal lobe, with one close to the primary motor cortex and another one more anterior. The table in Figure 1C shows the projection of each channel onto the brain cortex, following the nomenclature from Figure 1A.

To position the probe on the subjects’ head, we followed two different approaches: half of the subjects performed a standard approach and the other half followed a guided approach. In the standard approach, we adjusted the cap with a measuring tape following the standard metric procedure that considered the subject’s head circumference, nasion–inion, and ear-to-ear distances (Chatrian et al., 1985; Pfeifer et al., 2018). In the guided approach, the positioning of the fNIRS cap was based on the cortical anatomy. With a real-time neuronavigation system (section “Real-Time Neuronavigation”), a subset of optodes was first placed above the primary contralateral motor cortex. The position of the remaining optodes of the probe was constrained by the head cap. In both cases (i.e., standard and guided approaches), the position of each optode was captured with a commercial digitizer (Fastrak, Polhemus, Colchester, VT, United States). These positions were used for Monte Carlo simulations and for properly registering the position of optodes on a standard brain atlas. Due to time constraints, the subjects that were submitted to the guided approach performed three independent sessions in three different days, always at the same time of the day. In the standard approach, subjects performed five independent sessions: three sessions in three different days (to match the guided approach) and two extra sessions in one of the 3 days, at different times of the day (section “Subjects and Experimental Protocol”).

### Real-Time Neuronavigation

To perform the neuronavigation protocol in real time, we employed an in-house visualization system developed for this specific purpose (Rubianes Silva, 2017). A version runnable on Windows is freely available on our website^1^. First, subjects underwent an MRI session on a 3 T MRI scanner (Philips Achieva). The structural MRI image (T1-weighted with 1 × 1 × 1 mm^3^ resolution, TE = 3.2 s, TR = 7 ms, TI = 900 ms, 8° flip angle) was used as *a priori* information to guide the movements of the commercial digitizer on the subjects’ scalp during the fNIRS sessions. For this, a rigid transformation matrix that aligns the scanned volume with the corresponding physical head must be computed. Eight pairs of anatomical points (i.e., points from the physical head and its correspondence on the rendered scanned volume) must be provided manually. In this study, we used the inion, right outer corner of eye, nasion, preauricular left, preauricular right, glabella, left alar, and right alar as the anatomical references (Sforza and Ferrario, 2006; Ibrahim et al., 2016). The singular value decomposition (SVD) technique was applied to find the elements of the transformation matrix.

During the fNIRS sessions, the visualization system captures the position of the digitizer on the physical head, transforms it to the anatomical space of the MRI volume using the computed transformation matrix, and displays it as a triad cursor on the visible head’s surface. The setup to calibrate the neuronavigation space employs eight pairs of anatomical points (i.e., points from the physical head and its correspondence on the rendered scanned volume) that must be provided manually. In this study, we used the inion, right outer corner of eye, nasion, preauricular left, preauricular right, glabella, left alar, and right alar as the anatomical references (Sforza and Ferrario, 2006; Ibrahim et al., 2016). The depthmap-based picking algorithm was implemented to afford 3D interactions with rendered images (Wu et al., 2003, 2011). The reproducibility of the procedure was assessed through a one-way analysis of variance (ANOVA) of a series of measurements of randomly chosen seven points on different heads at different instants of time. It only failed when the subject did not have palpable inion.

With the visualization system described above, we used the MRI image to guide the placement of the digitizer over the primary motor cortex at all sessions of each subject (Yousry et al., 1997). In the first session of measurements, we saved the position of the fNIRS optodes that were located above the motor cortex. In the subsequent sessions, small spheres with a radius of less than 6 mm were displayed on these saved positions to guide the placement of optodes. This ensured that all the optodes were placed very close on the head across different sessions.

### NIRS Preprocessing

After the fNIRS data collection, we first removed channels with a low signal-to-noise ratio (SNR), defined as SNR < 8. We computed the SNR as the mean intensity of each channel divided by the standard deviation of the same channel, and the threshold was chosen based on previous works (de Oliveira et al., 2017, 2019; Forero et al., 2017; Novi et al., 2018). Next, we converted light intensity at each wavelength of each channel to optical density (OD) and removed motion artifacts with spline interpolation followed by wavelet decomposition (Scholkmann et al., 2010; Molavi and Dumont, 2012; Di Lorenzo et al., 2019; Novi et al., 2020). Hemoglobin concentration changes were estimated using the modified Beer–Lambert law with a pathlength factor equal to six for both wavelengths (Huppert et al., 2009b). Finally, we filtered the concentration changes with a bandpass filter between 0.005 and 0.5 Hz in order to remove low-frequency drifts, HR, and other high-frequency physiological noise (Joseph et al., 2006; Pinti et al., 2019).

### Test–Retest Reliability Analyses

To investigate the reproducibility of the fNIRS signal, each channel was classified as activated or not activated. A channel was assigned as activated if it presented a characteristic hemodynamic response (i.e., significant increase in oxy-hemoglobin and decrease in deoxy-hemoglobin during the task period compared to the baseline period). The dynamics of the hemodynamic response was inferred by a general linear model (GLM) with a gamma function (Santosa et al., 2019). For each long fNIRS channel, the closest short-channel measurement was incorporated in the GLM model as a regressor to remove extra-cortical hemodynamics (Gagnon et al., 2012; Goodwin et al., 2014; Yücel et al., 2015; Sato et al., 2016). The GLM was solved with an iterative algorithm introduced by Barker et al. (2013). The algorithm uses an autoregressive model based on pre-whitening filter and robust regression to remove both serially correlated errors and remaining motion artifacts. Channels that presented significant responses (*p* < 0.05) for *both* oxy-hemoglobin and deoxy-hemoglobin were classified as activated (Huppert, 2016; Pinti et al., 2017) with the additional constrain that the coefficient β associated with the gamma function needed to be positive for HbO (β_*H°b°O*_ > 0) and negative for HbR (β_*H°b°R*_ < 0). By simultaneously analyzing HbO and HbR time series and using the pre-whitening filter, we decreased false-positive rates in the hemodynamic response function (HRF) (Kirilina et al., 2012; Barker et al., 2013).

Standard group analysis was performed with the coefficients (β_*HbO*_ and β_*HbR*_) found after solving the GLM model for each channel of each volunteer. To compute the beta coefficients for a given group of volunteers ($\beta_{H\,b\,O}^{g}$ and $\beta_{H\,b\,R}^{g}$, where *g* stands for group), we performed a weighted-linear regression to correct different errors of β-values of each channel and each measurement.

To assess the reproducibility across different sessions, we obtained a binary vector, *B^s^*, for each session of each subject from the GLM. The *j*th element of the *B^s^* vector, $B_{j}^{s}$, was assigned to 1 if the *j*th channel from a session *s* had a characteristic hemodynamic response (β_*H°b°O*_ > 0 and β_*H°b°R*_ < 0 with *p* < 0.05); otherwise, the element was set to 0. With the binary vectors, we assessed the spatial within-subject reproducibility of a given joint of channels with two metrics that have been previously used in the fNIRS literature (Plichta et al., 2007; Schecklmann et al., 2008; Blasi et al., 2014). The metrics compare the size (*R*_*q*_) and the overlap (*R*_*o*_) of the evoked hemodynamic response over different sessions:

$$R_{q}^{i,j}=1-\frac{\left|A_{s_{i}}-A_{s_{j}}\right|}{A_{s_{i}}+A_{s_{j}}},$$

$$R_{o}^{i,j}=2\times \frac{A_{o\,v\,e\,r\,l\,a\,p}^{i,j}}{A_{s_{i}}+A_{s_{j}}},$$

where *A*_*s_i*_ is the number of activated channels of session *i* and $A_{o\,v\,e\,r\,l\,a\,p}^{i,j}$ is the number of common activated channels in sessions *i* and *j*. To verify the channel-wise within-subject reproducibility, we created frequency vectors that show the number of times that each channel was activated over different sessions for a given subject (de Oliveira et al., 2017, 2019; Novi et al., 2020). A high frequency of a given channel means that this channel was recruited over different sessions repeatedly.

## Results

### Group Results Evidence Robust Contralateral Activation

**FIGURE 2:**
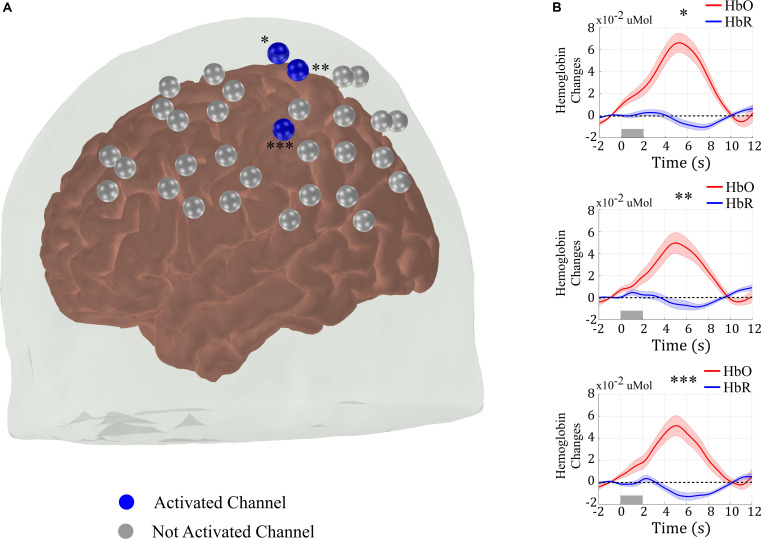
**(A)** Results from the group analysis using the measures from all five sessions of the five subjects acquired with the standard approach (i.e., without the assistance of a neuronavigation system) to position the optical probe. The right-brain hemisphere did not have any activated channel in the group analysis. **(B)** Evoked hemodynamic response of the activated channels in **(A)**. The hemodynamic response was computed by block-averaging the measured fNIRS signal across all sessions for all volunteers of the blue channels shown in **(A)** and marked with asterisks.

Figure 2A shows the group response to the right finger-tapping stimulation in the five subjects acquired with the standard protocol (i.e., without the neuronavigation system and with a total of 25 measurements). As expected for a motor stimulation, the right finger-tapping task induced significant hemodynamic changes in the channels located on the contralateral motor cortex (Obrig et al., 2000; Franceschini et al., 2003). In addition to the spatial analysis, we also computed the measured HRF of the activated channels by averaging the concentration changes across all trials for the same subject, and then averaging the subject HRF across all volunteers (Figure 2B). As compared to the baseline, the measured HRF of each activated channel is temporally well-behaved, presenting a clear increase in oxy-hemoglobin (HbO) and a decrease in deoxy-hemoglobin (HbR) concentration synchronized with the period of stimulation. These results show that we were able to recover the expected response to a simple motor stimulation task even with a small sample size. However, this result does not inform about the consistency of activation across different subjects or the reproducibility of the activation for the same subject.

### Individual fNIRS Response Is Highly Variable in the Standard Approach

To analyze the reproducibility of the fNIRS response at the within-subject level, we quantified the frequency of activation of each channel for each subject acquired with the standard approach. The first interesting finding that contrasts with the group analysis concerns the location of the hemodynamic response. Although the HRF was well localized in the contralateral primary motor cortex for the group, the number of channels that were activated at least once across all sessions for the same subject ranged from 0 to 65% of the whole probe. Despite the fact that the probe was entirely located around the somatomotor cortex, the heterogeneity of the response evidences the individual variability of the fNIRS results, as pointed by previous studies (Plichta et al., 2007).

The low reproducibility of the fNIRS signal in the standard approach was quantified by the values of *R*_*q*_ and *R*_*o*_ (Table 1). To evaluate these two parameters for the data acquired with the standard approach, we separated the sessions performed on the same day and the sessions performed across different days. For the sessions acquired on the same day, the median *R*_*q*_ and *R*_*o*_ across all subjects were 0.73 and 0.36, respectively. The reproducibility was lower for the sessions acquired across different days: 0.66 and 0.04 for *R*_*q*_ and *R*_*o*_, respectively.

**TABLE 1:** Reproducibility parameters (*R*_*q*_ and *R*_*o*_) for the data acquired with the standard protocol, along with variations in heart rate (ΔHR) and mean arterial pressure (ΔMAP).

	Same day				Across days			
		
Subject	ΔMAP (%)	ΔHR (%)	*R*_*q*_	*R*_*o*_	ΔMAP (%)	ΔHR (%)	*R*_*q*_	*R*_*o*_
1	3.1	11.8	0.00	0.00	8.3	8.9	0.00	0.00
2	14.2	12.5	0.41	0.17	5.1	6.8	0.13	0.00
3	5.5	20.7	0.73	0.45	8.3	14.5	0.66	0.31
4	13.2	6.8	0.89	0.36	8.3	4.0	0.71	0.04
5	4.7	9.5	0.9	0.68	5.1	9.0	0.94	0.71
**Median**	5.5	11.8	0.73	0.36	8.3	9.0	0.66	0.04

*Both *R*_*q*_ and *R*_*o*_ were computed for the whole probe.*

Specifically in the contralateral primary motor cortex, which is the expected activated region for the motor task, channels in this region were activated in all sessions for only one subject, regardless of the protocol (i.e., sessions on the same day or sessions across different days). Although most of the channels of the probe were activated at least once over the different sessions, the left brain hemisphere was not activated in eight out of the 25 sessions for the data acquired with the standard approach.

### Systemic Physiology Does Not Explain All Variability in fNIRS Data

**FIGURE 3:**
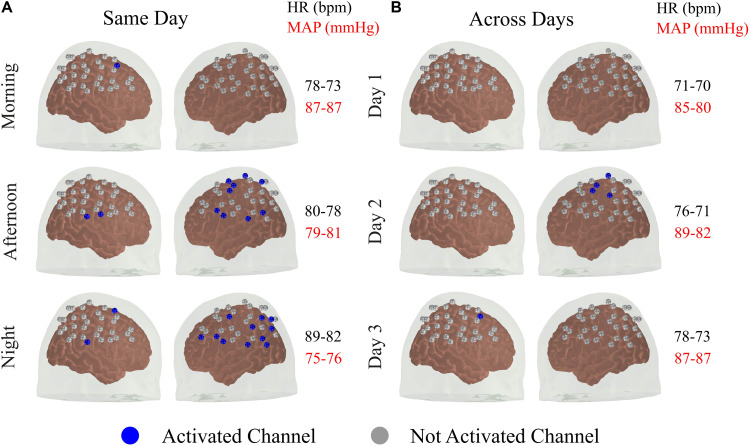
Activated brain regions for one representative subject over different sessions **(A)** within the same day and **(B)** across subsequent days. A channel was classified as activated for a *p* < 0.05 for both HbO and HbR with the additional constrain that the coefficient associated with HbO and HbR needed to be positive and negative, respectively. In addition to the activated brain regions in each session, the values of heart rate (HR) and mean arterial blood pressure (MAP) acquired immediately before and after the fNIRS measurements are shown.

From Section “Individual fNIRS Response Is Highly Variable in the Standard Approach,” it appears that the reproducibility of the fNIRS response is slightly higher for sessions acquired at different times on the same day, compared to the sessions performed at the same time of the day for different days. This led us to further investigate the extent to how much of the within-subject variability could be associated with systemic physiology (and, in particular, the circadian cycle) for the data acquired with the standard approach. Figure 3 shows the activated fNIRS channels along with the systemic physiological parameters for a representative subject in each session with the standard approach. As expected, both HR and mean arterial blood pressure (MAP) oscillated during the different measures taken over a single day, likely due to the circadian cycle (Figure 3A). For the subject in Figure 3, HR and MAP varied 12.5 and 14.2% across morning, afternoon, and evening measurements, respectively. The fNIRS activation pattern also varied within the sessions of the same day. The average (standard error) fNIRS reproducibility indexes were *R*_*q*_ = 0.41 (0.31) and *R*_*o*_ = 0.17 (0.20) for this specific subject. A significant fNIRS response in the contralateral primary motor cortex was found in two out of the three sessions performed on the same day. Considering all subjects, the median HR and MAP variations in the same day were 11.8 and 5.5%, respectively, while the median *R*_*q*_ and *R*_*o*_ were 0.73 and 0.36, respectively.

For the measurements performed at the same time of the day in different days, the circadian cycle is expected to have less influence on systemic physiology; therefore, variations in HR and MAP are expected to be smaller. For the subject in Figure 3, the average changes in HR and MAP were 6.8 and 5.1%, respectively, while the average (standard error) fNIRS reproducibility indexes were *R*_*q*_ = 0.13 (0.16) and *R*_*o*_ = 0.00 (Figure 3B). Considering all subjects, we observed a median HR and MAP change by 9.0 and 8.3%, respectively, with median *R*_*q*_ and *R*_*o*_ of 0.66 and 0.04, respectively. Table 1 summarizes the reproducibility metrics and variations in HR and MAP for all subjects.

Interestingly, although the variations of HR and MAP were similar for measurements on the same day compared to inter-day measurements, the size and spatial overlap of the hemodynamic responses were more reproducible on the same day rather than across different days. This last finding, along with the fact that we performed short-channel regression, suggests that part of the variability in the fNIRS signal may not come from global physiological noise.

As an attempt to quantify the effects of systemic physiology in the variability of the fNIRS signal, we further computed the complement of *R*_*q*_ (i.e., ν_*q*_≡1−*R*_*q*_) as a measure of variability instead of reproducibility of the fNIRS results. By assuming a linear relationship between fNIRS variability and both HR and MAP changes (i.e., < *v*_*q*_≥α + βΔ*H°R* + γΔ*M°A°P*), we performed a regression analysis to quantify the degree of dependency of the fNIRS variability on the systemic variables. We found that neither HR nor MAP changes can predict fNIRS variability. The fit estimates (standard error, *p*-value) for α,β, and γ were, respectively, 0.62 (0.47, *p* = 0.23), −0.37 (3.0, *p* = 0.90), and −1.6 (3.8, *p* = 0.61). The low significance of the contribution of HR and MAP to the fNIRS variability suggests that there may be other confounding factors that drive the intra-subject variability in fNIRS data.

### Guided fNIRS Experiments Present Lower Within-Subject Variability

Last, we hypothesized that the variation on the spatial location of the fNIRS optodes across different sessions may contribute to the fNIRS variability. To reduce the spatial variation in probe positioning, we employed a real-time neuronavigation system to reliably position the fNIRS optodes above the motor cortex and to ensure the reproducibility across the sessions (see section “Real-Time Neuronavigation”). To properly compare the reproducibility of the guided and the standard approaches, we considered only the sessions that were acquired at the same time across different days. Each subject performed three independent sessions with this condition in both approaches.

**FIGURE 4:**
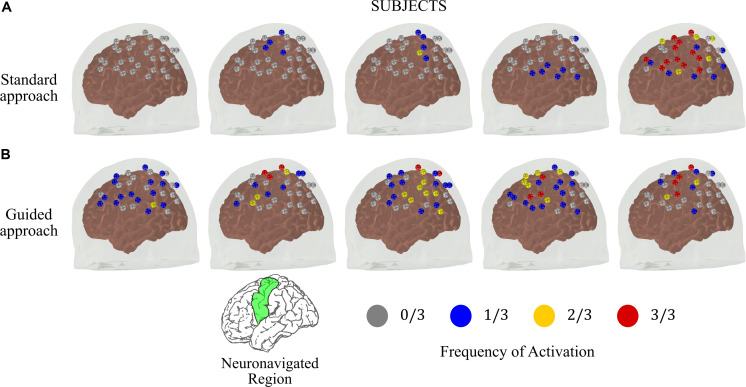
Frequency of activated brain regions at the contralateral brain hemisphere due to right finger-tapping at the subject level for the measurements performed at the same time across different days (total of three sessions) for the **(A)** standard and **(B)** guided approaches. The green region in the reference brain (located at the bottom of the figure) corresponds to the targeted brain region that we used as reference to position the optical probe in the guided approach.

Figure 4 shows the frequency maps of activation on the left hemisphere for each subject. The differences in reproducibility between the two approaches are clear. Most of the subjects acquired with the standard approach did not have activated channels in the contralateral hemisphere with frequency higher than 1/3 (Figure 4A). On the other hand, the guided approach showed robust and consistent activation maps in the contralateral motor cortex. Activation on the same channels was repeated at least twice for all subjects that performed the guided protocol. In addition, at least one channel in the contralateral motor cortex was activated at all sessions in four of the five subjects.

We also compared the inter-session measures of size (*R*_*q*_) and spatial overlap (*R*_*o*_) from both approaches. Table 2 summarizes the results obtained for all volunteers when *R*_*q*_ and *R*_*o*_ were calculated for the whole probe, for the left hemisphere, and for the neuronavigated region. The reproducibility of the guided approach exceeded the standard approach in all situations. When considering all channels of the probe, we observed reproducibility medians of 0.66 (*R*_*q*_) and 0.04 (*R*_*o*_) with the standard approach, while the guided approach yielded 0.71 (*R*_*q*_) and 0.36 (*R*_*o*_). If one considers only the contralateral brain hemisphere (i.e., left hemisphere), the size and the overlap of the evoked hemodynamic response decrease in the standard approach (*R*_*q*_ = 0.13 and *R*_*o*_ = 0.00) while they both remained approximately constant in the guided approach (*R*_*q*_ = 0.69 and *R*_*o*_ = 0.43). These trends continue as one analyzes the activation reproducibility more locally. Considering only the neuronavigated brain region, we observed median *R*_*q*_ equals to 0.17 and 0.70, and *R*_*o*_ equals to 0.00 and 0.59 for the standard and guided approaches, respectively. On average, the neuronavigation system increased the reproducibility in size and spatial overlap of the fNIRS signal by a factor of two.

**TABLE 2:** Comparison of the fNIRS signal reproducibility for the data acquired with the standard and guided approaches.

	Whole probe				Left hemisphere				Neuronavigated region			
			
Subject	*Standard*		*Guided*		*Standard*		*Guided*		*Standard*		*Guided*	
						
	*R*_*q*_	*R*_*o*_	*R*_*q*_	*R*_*o*_	*R*_*q*_	*R*_*o*_	*R*_*q*_	*R*_*o*_	*R*_*q*_	*R*_*o*_	*R*_*q*_	*R*_*o*_
1	0.00	0.00	0.54	0.08	0.00	0.00	0.69	0.07	0.00	0.00	0.13	0.00
2	0.13	0.00	0.71	0.51	0.13	0.00	0.63	0.56	0.17	0.00	0.70	0.70
3	0.66	0.31	0.34	0.32	0.11	0.11	0.39	0.37	0.17	0.17	0.44	0.44
4	0.71	0.04	0.75	0.36	0.29	0.00	0.82	0.43	0.00	0.00	0.85	0.59
5	0.94	0.71	0.9	0.49	0.94	0.77	0.83	0.65	0.89	0.89	0.87	0.73
**Median**	0.66	0.04	0.71	0.36	0.13	0.00	0.69	0.43	0.17	0.00	0.70	0.59

*The numbers show the size (*R*_*q*_) and the overlap (*R*_*o*_) of the evoked hemodynamic response for all channels (whole probe), for the left hemisphere, and for the neuronavigated region. In all situations, the reproducibility of the guided approach was higher than the standard approach.*

## Discussion

In this work, we aimed to investigate the reproducibility of fNIRS measures. Since fNIRS has rapidly expanded its applications in functional neuroscience, the importance of obtaining reproducible results has also grown. Therefore, test–retest studies have become a topic of debate in the fNIRS community. Here, by collecting functional motor stimulation data from 10 healthy subjects across multiple sessions on different days and during the same day, we characterized the inter- and intra-subject reproducibility of a simple and predictive task: block-designed finger tapping.

In line with previous reports, our results show that finger-tapping stimulation induces a robust fNIRS response in the contralateral primary motor cortex with the standard approach of positioning the optodes, even for a small group of subjects (Figure 2). Compared to baseline, HbO (HbR) significantly increased (decreased) in the region of interest during stimulation, just as expected. However, when we compared the individual responses to the task, it is possible to observe a wide variation in the activation maps (Figures 3, 4). The low reproducibility of fNIRS was also quantified by measuring the size and spatial overlap of the evoked hemodynamic responses over different sessions (Tables 1, 2). For the three independent sessions performed in the same day, the median *R*_*q*_ and *R*_*o*_ were 0.73 and 0.36, respectively, considering all volunteers. Independent sessions performed during the same time of the day at different days yielded an even lower reproducibility (*R*_*q*_ = 0.66 and *R*_*o*_ = 0.04) across all volunteers, despite the smaller variations in global systemic physiology under this condition.

Several factors can influence the reproducibility of fNIRS results. From the methodological perspective, it is important to combine oxy- and deoxy-hemoglobin data to decide whether a channel presented a characteristic hemodynamic response (i.e., an increase in oxy-hemoglobin and a decrease in deoxy-hemoglobin during the period of stimulation compared to the baseline period). Although this is the most basic approach in order to minimize false-positive rates of activation, most studies still report only changes in one of the chromophores. Specially for the HbO change, it is highly susceptible to arteriolar vessels, which are more responsive to systemic physiological changes (Huppert et al., 2009b; Tachtsidis and Scholkmann, 2016). For example, an increase in cerebral blood volume can induce an increase in both oxy- and deoxy-hemoglobin; motion artifacts can also change HbO and HbR concentrations. In our data, we verified that if we did not evaluate both HbO and HbR, we would have incorporated false positives to our analysis, which increases the variability at both the inter- and intra-subject levels. Since these contributions are expected to be random across sessions and subjects, they tend to average out at the group level, which explains the higher reproducibility of the fNIRS-based results for a group of subjects.

Another confounding factor in the fNIRS signal comes from hemodynamic contaminations from the extra-cortical layers and systemic physiology. In this work, we proposed to remove extra-cortical contributions by performing additional measurements with four short channels located on the primary and secondary motor cortices (Gagnon et al., 2012). Recent studies have demonstrated that removal of global noise leads to a more accurate localization in the hemodynamic response (Yücel et al., 2015; Dravida et al., 2017). While the localization of the hemodynamic response reduces the number of activated brain regions due to a single task, it does not decrease the spatial variability of the fNIRS signal at the channel level. In fact, the reduction in the number of activated channels may increase the channel inter- and intra-subject variability due to the lack of accuracy in repositioning the optical probe at several sessions. Therefore, a channel-wise comparison (such as the ones performed with GLM) may fail to find real activated regions.

Although short-channel regression can reduce the influence of global systemic physiology in the fNIRS signal, previous studies have shown that it is not completely effective (Kirlilna et al., 2013). Indeed, our data suggest that there is a weak to moderate correlation between fNIRS variability and global systemic physiology (as measured by HR and MAP) even after removing extra-cortical contributions (Figure 3 and Table 1). In our work, we observed that the highest variation of systemic physiology occurred in the measurements performed on the same day (as expected due to the circadian cycle). However, the values of the metrics of reproducibility (*R*_*q*_ and *R*_*o*_) were on average twice as high for the data acquired on the same day than for the data acquired in different days with the standard approach. This result suggests that part of the variability in the fNIRS signal does not come from physiological contamination, only. Unfortunately, in this work, we measured HR and MAP at the beginning and end of each session. Continuous monitoring of systemic physiology simultaneous with fNIRS may evidence a greater relationship between HR/MAP and fNIRS variability than the ones reported in this work (Caldwell et al., 2016).

The proper positioning of fNIRS sources and detectors are usually neglected with the argument that the intrinsic spatial resolution of the technique is poor by itself. By neglecting this uncertainty, it has been widely assumed that systemic and extra-cortical physiological noise triggered by the task can explain all variability presented in the fNIRS data (Blasi et al., 2014; Dravida et al., 2017). In this work, we provide a robust methodological approach to constrain the spatial variability in fNIRS protocols and to acquire missing anatomical information with a real-time navigation procedure. Our approach requires *a priori* information from a high spatial resolution imaging that carries brain anatomy information of each volunteer. Here, we opted to use structural MRI images, but other neuronavigation systems (as well as ours) can also work with computed tomography (CT) scans (Rubianes Silva, 2017; Souza et al., 2018, 2019). The validation of our approach shows that by adding spatial information to the protocol, we were able to improve the within-subject reproducibility of the fNIRS results by an average factor of almost three in the region of interest (Table 2). More importantly, the guided approach with the neuronavigator was sensitive to functional activation in every session of every subject. Although the low number of subjects limited statistical inferences, results obtained with our proposed neuronavigation protocol suggest that imprecise positioning of fNIRS optodes is an important source of variability in fNIRS that needs to be taken into account.

## Conclusion

In the present work, we observed that guided measurements with real-time anatomical information can significantly improve the within-subject reproducibility of the fNIRS results. By performing a functional finger-tapping protocol, we found that the within-subject fNIRS reproducibility increased by a factor of two on average. The guided approach also increased the sensitivity of fNIRS measurements to detect the evoked hemodynamic response induced by the finger-tapping task. While the standard experimental approach yielded heterogeneous activation patterns that vary across subjects and sessions for the same subject, every session acquired with the assistance of the neuronavigation system presented a characteristic hemodynamic response in the contralateral brain hemisphere for all subjects. Although more data are needed to extend the validity of the observed results, the present work brings attention to the fact that proper probe positioning may play an important role in the intra-subject reproducibility of the fNIRS signal.

## Data Availability Statement

The datasets generated for this study are available on request to the corresponding author.

## Ethics Statement

The studies involving human participants were reviewed and approved by the Ethics Committee at the University of Campinas. The patients/participants provided their written informed consent to participate in this study.

## Author Contributions

SN and EF performed the data acquisition, analyzed the data, and wrote and edited the manuscript. NS performed the data acquisition and contributed to the data analysis. JR and S-TW edited the manuscript and contributed to the design and development of the neuronavigation system. AQ and GM contributed on designing the experimental protocol. RM conceived the main idea of the work (including the hypothesis), supervised the data analysis, and edited and wrote the manuscript. All authors discussed the results and contributed to the final edition of the manuscript.

## Conflict of Interest

The authors declare that the research was conducted in the absence of any commercial or financial relationships that could be construed as a potential conflict of interest.

## Footnotes

1 http://www.light4brain.com
